# The Association between Community Water Fluoridation and Bone Diseases: A Natural Experiment in Cheongju, Korea

**DOI:** 10.3390/ijerph17249170

**Published:** 2020-12-09

**Authors:** Naae Lee, Sungchan Kang, Woojoo Lee, Seung-sik Hwang

**Affiliations:** Department of Public Health Sciences, Graduate School of Public Health, Seoul National University, Seoul 08826, Korea; jenni0428@snu.ac.kr (N.L.); scknag81@snu.ac.kr (S.K.); lwj221@snu.ac.kr (W.L.)

**Keywords:** community water fluoridation, adverse health effect, bone diseases, natural experiment, spatio-temporal analysis

## Abstract

The present study aimed to investigate the association between bone diseases and community water fluoridation (CWF). An ecological study with a natural experiment design was conducted in Cheongju, South Korea, from 1 January 2004 to 31 December 2013. The community water fluoridation program was implemented in Cheongju and divided into CWF and non-CWF areas. To observe adverse health effects related to bone diseases, we conducted a spatio-temporal analysis of the prevalence of hip fracture, osteoporosis, and bone cancer in residents who have lived in CWF and non-CWF areas using National Health Insurance Service data. First, we used standardized incidence ratios to estimate the disease risk. Second, the hierarchical Bayesian Poisson spatio-temporal regression model was used to investigate the association between the selected bone diseases and CWF considering space and time interaction. The method for Bayesian estimation was based on the R-integrated nested Laplace approximation (INLA). Comparing the CWF area with the non-CWF area, there was no clear evidence that exposure to CWF increased health risks at the town level in Cheongju since CWF was terminated after 2004. The posterior relative risks (RR) of hip fracture was 0.95 (95% confidence intervals 0.87, 1.05) and osteoporosis was 0.94 (0.87, 1.02). The RR in bone cancer was a little high because the sample size very small compared to the other bone diseases (RR = 1.20 (0.89, 1.61)). The relative risk of selected bone diseases (hip fractures, osteoporosis, and bone cancer) increased over time but did not increase in the CWF area compared to non-CWF areas. CWF has been used to reduce dental caries in all population groups and is known for its cost-effectiveness. These findings suggest that CWF is not associated with adverse health risks related to bone diseases. This study provides scientific evidence based on a natural experiment design. It is necessary to continue research on the well-designed epidemiological studies and develop public health prevention programs to help in make suitable polices.

## 1. Introduction

Community water fluoridation (CWF) aims to prevent dental caries by adjusting the amount of fluoride in tap water to ensure that it is fit for human consumption [1]. Considering the fact that care for oral health presents a significant burden to national health, CWF is the most economical and effective public health care method to prevent tooth decay even among adults [2,3]. Health authorities, including many researchers, have argued that if CWF is meant to benefit the large population, it is necessary to assess all the risks associated with it. Consequently, many epidemiological studies are needed to assess its risk, yet only a few people consume fluoridated water for a long time because some people are against CWF. This makes it difficult to conduct research on the effects of CWF and sets off a complicated argument.

Proponents of CWF have argued that it is an important public health project that is safe and an effective method to prevent dental caries, and is beneficial everyone, regardless of age, income, or gender (especially younger members of society) [4,5]. Conversely, opponents claim that fluoride is toxic and emphasizes the potential for various adverse effects [6]. Moreover, children may experience excessive exposure to fluoride because parents do not educate them about the effects of CWF and the amount of toothpaste they need to use [7]. Other problems associated with CWF include environmental destruction caused by fluoridated water [8], negative effects of various diseases caused by drinking fluoridated water (dental fluorosis, cancer, and IQ) [9], and violation of an individual’s right to choose. Open debates are raging globally due to conflicting claims from both parties [10], and various results based on the pros and cons of CWF have been published.

Statistics from the Ministry of Health and Welfare of the Republic of Korea indicate that CWF began in 1981, in Jinhae, and gradually expanded to about 37 water purification plants (29 local authorities) in 2002 [11]. As the CWF program has flourished, many research papers on its benefits for dental caries have been published in Korea [12,13,14]. A recent study showed that CWF decreased caries in permanent teeth in cities where CWF was conducted for 17 years [15]. Though similar to the international case, in Korea, the debate over CWF has not been concluded because of the above reasons. Due to this growing opposition, CWF was being operated in only 14 water purification plants (10 local authorities) in December 2017, but in December 2018, the implementation of CWF projects completely ceased in Korea.

In recent years, there are few studies investigating the intellectual abilities of children who were exposed to fluoride during pregnancy [16] or exposure during infancy in children who were breast-fed or formula-fed [17] or the association between an increase in the amount of fluoride in tap water by 1 mg/L and higher odds of an ADHD diagnosis [18]. There is a lack of research focusing on the harmful effects of fluoride exposure in other kinds of diseases. In terms of the hazardous effects caused by fluoride exposure, there are few studies related to bone diseases such as hip fractures or bone density [19,20], and outcomes of most studies have been reported to be irrelevant to CWF projects, and there is still insufficient research on the harm caused by fluoride exposure.

In this study, we investigated the effect of drinking tap water by comparing hip fractures, osteoporosis, and bone cancer prevalence in Cheongju, South Korea, where the area was naturally divided depending on the implementation status of drinking fluoridated water.

## 2. Study Area and Data

### 2.1. Study Design and Locations

**Figure 1:**
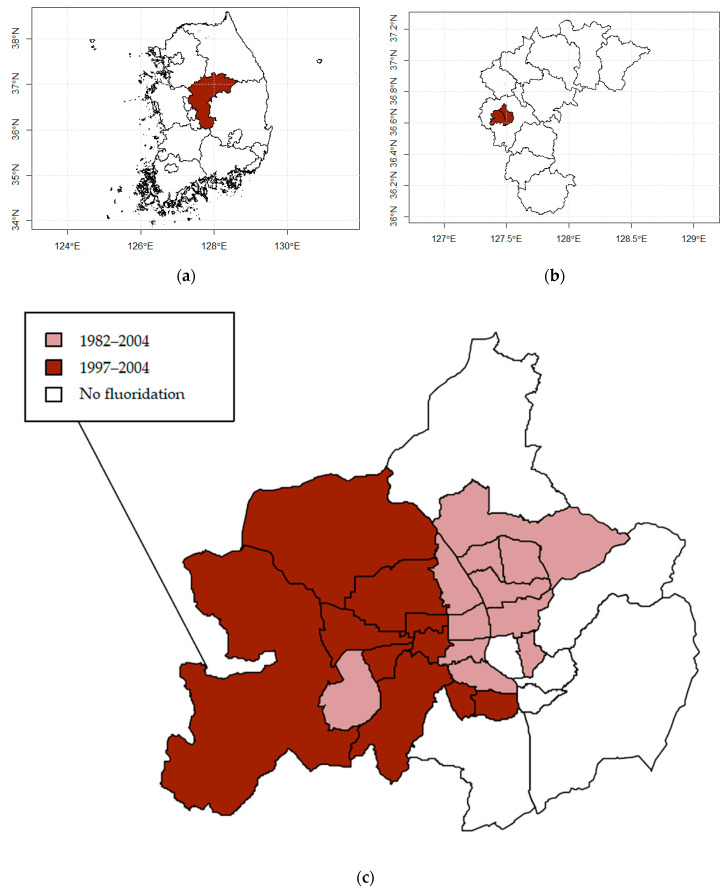
Community water fluoridation implementation. (**a**) Map of the Republic of Korea and the dark red area represents the Chungcheong province. (**b**) Map of Cheongju city in the province of Chungcheong, (**c**) Map of fluoridated areas and non-fluoridated areas in the community with different time periods.

This is an ecological study with a natural experiment design. Although there are numerous areas in Korea where CWF was conducted, we chose Cheongju city in Chungcheong province as our study area (Figure 1a,b). In this area, the number of towns can be statistically comparable, and the time CWF was implemented was sufficient to observe the health effects. In addition, this area was naturally been divided into two regions: fluoridated areas and non-fluoridated areas (Figure 1c)). The area colored in dark red is the area where the CWF was conducted from 1982 to 2004, whereas the area colored in dark pink is where CWF was conducted from 1997 to 2004. In the white area, CWF was never conducted. In the same population, this study area had appropriate conditions to examine the health effects of CWF. Cheongju consists of 28 towns, and residents are supplied with tap water from local water purification plants. Youngun and Jibuk water treatment plants supply 21 towns with fluoridated tap water and 7 towns with non-fluoridated tap water.

### 2.2. Data Sources and Variables

In our study, we examined the adverse health effects of fluoridated tap water compared with those of non-fluoridated tap water in residents of the Cheongju region from 2004 to 2013, using the National Health Insurance Service (NHIS) data. The NHIS is considered a major institution for the management of all Korean citizens’ medical expenses for medical claims [21]. The reason we used the NHIS data was to provide representative data on all Korean citizens, and 98% of the population is covered by this national insurance [22]. Since we requested customized data from the NHIS, there was no missing information, and we obtained each of the selected diseases in the form of frequency by gender (male vs. female), year (2004–2013), age ranges (0–9, 10–19, 20–29, 30–39, 40–49, 50–59, 60–69, 70–79, and 80 above) for each year, and address in town level. 

The study population included all residents in Cheongju, and we used the ICD-10 code for selected bone diseases: hip fracture (S72), osteoporosis (M80–82), and bone cancer (C40–41). To observe the general characteristics of residents who live in fluoridated and non-fluoridated areas, we used general variables such as gender, age, population density, and the number of towns. For the variables related to exposure to fluoridation, we used the period of residence, source of water, and types of drinking water from the Korean Microdata Integrated Service (MIDS) of Statistics Korea.

First, we computed descriptive statistics of residence in Cheongju to assess the general characteristics of residents in Cheongju. To compare rates between two geographical areas, we implemented the direct method of standardization using the 2010 Population and Housing Census from Statistics Korea as the standard population.

## 3. Methodology

### 3.1. Modeling Health Risk

In order to apply Bayesian spatio-temporal regression analysis, we calculated the expected count data by applying the standard population. Then, we measured the disease risk by calculating standardized incidence ratios (SIRs) per 10,000 person–years. The SIR can be calculated by an observed count in each areal unit as *i* expressed as *Y* = (*Y*_1_, *…*, *Y_n_*) and a set of expected disease counts in area *i* is given by *E* = (*E*_1_, …, *E_n_*). These expected counts were calculated based on the age and sex within each areal unit and are expressed as follows:

(1) $${\;\mathrm{SIR}}_{i}=\frac{Y_{i}}{E_{i}}$$

### 3.2. Spatio-Temporal Model

Data on selected diseases were collected according to space and time, and there may be space and time relatedness. Therefore, in our study, we applied the spatio-temporal model to explain the dependence of space and time correlation, and the conditional autoregressive (CAR) model was applied to control spatial correlation. With the disease count data for each small area in Cheongju (town unit, *i* = 1, …, 28) and time period from 2004 to 2013 (*t* = 1, …, 10), the model can be expressed in the Poisson distribution form of each area and time coefficient.

(2) $$y_{it}\;\sim \;Poisson\left(E_{it}\mu_{it}\right),\;i=1,\ldots ,n,\;t=1,\ldots T\;E\left(y_{it}\right)=\;\lambda_{it}=e_{it}\mu_{it}\;\log\left(\mu_{it}\right)=\;x_{it}^{T}\beta +\mu_{i}+v_{i}+\gamma_{t}+\delta_{it}$$

where $x_{ij}^{T}\beta$ is an overall risk level, $\mu_{i}$ represents the spatial level for the *i*-th area (town unit, *i* = 1, …, 28), which considers the spatial correlation.$\;v_{i}$ represent for unexplained spatial correlation and $\gamma_{t}$ represents temporal effects from 2004 to 2013, and $\delta_{it}$ represents space × time interaction. 

We fitted four types of interactions using the Kronecker product proposed by Knorr-Held [23]. For Type 1 interaction, if two unstructured main effects $v_{i}\;\mathrm{and}\;\gamma_{t}$ are expected to interact, then space-time interaction $\delta_{it}$ are a priori independent. Type 2 interaction expect to interact between $\mu_{i}$ and $\gamma_{t}$, then each $\delta_{it}$ follows random walk independently. In the case of Type 3, the main effects $\mu_{i}$ and $\gamma_{t}$ interact, then $\delta_{it}$ follows an independent intrinsic autoregression. Finally, Type 4 interaction yields two dependent main effects have interaction, so $\delta_{it}$ is dependent over space and time. Comparisons between models used a deviance information criterion (DIC) to select a model with a smaller DIC value, where $\bar{D}$ is the mean posterior deviance and $p_{d}$ is the effective number of parameters. The DIC equation can be defined as

(3) $$DIC=\;\bar{D}+p_{d}$$

The parameters used for each type and the DIC obtained using R-INLA were expressed for each disease. Formulas from Type 1 to Type 4 interactions used in R-INLA are added to the supplementary file. Among the four interactions described above, the more suitable was selected as Type 1 according to the principle that it had a smaller DIC value. In the case of osteoporosis, type 2, 3, and 4 were too large, and for bone cancer, type 3 had a small DIC value. Overall, Type 1 interaction was the most appropriate. 

### 3.3. Sensitivity Analysis

In this study, the sensitivity analysis was performed through five methods using the CARBayesST package with Markov-chain Monte Carlo (MCMC) sampling to identify the interactions of space–time interaction. Sensitivity analysis can explain similarities and differences between models. CARBayesST is the first proprietary software package for spatio-temporal unit modeling with CAR [24]. Five models were applied using CARBayesST, and the code for CARBayesST was attached to the Supplementary Materials. The quality of the model fit was defined using DIC (Equation (3)). The model with the lowest DIC was considered to provide the best fit to the observed data [25]. We used STATA version 16.0 and R version 3.6.1 for data analysis. 

### 3.4. Ethics Approval

This study was approved by the Institutional Review Board (IRB) of Seoul National University (E1903/003-006).

## 4. Results

Table 1 represents the general characteristics of the participants divided by fluoridated and non-fluoridated areas. All variables (except population density and number of towns) were expressed as frequency and percentage (%). The population was approximately twice as high in fluoridated areas, but the gender distribution was almost the same in both areas. By age group, 20–39 years old (33.4%) were the highest group in the fluoridated areas and 40–59 years old (35.8%) were the highest in the non-fluoridated areas. The frequency of residence (those who have lived in the fluoridated areas for more than 25 years) was slightly higher for fluoridated areas than non- fluoridated areas (CWF = 9.01% and non-CWF = 5.13%). In both regions, drinking tap water had the highest proportion (CWF = 47.8% and non-CWF = 45.5%). This indicates that both regions were exposed to tap water at a similar rate. Generally, there were no regional differences between fluoridated areas and non-fluoridated areas.

**Figure 2:**
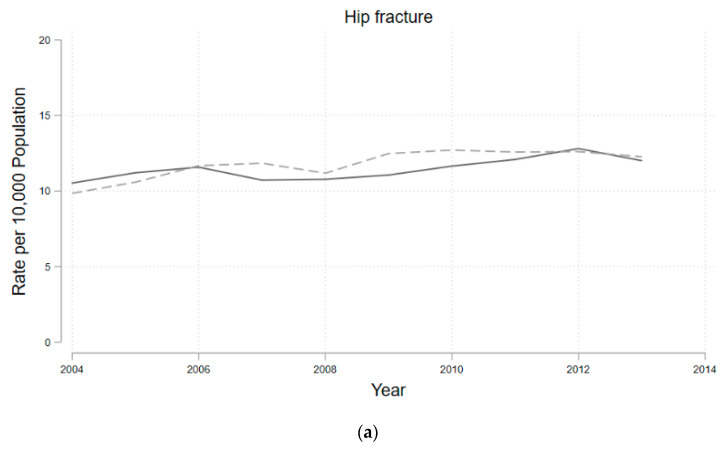

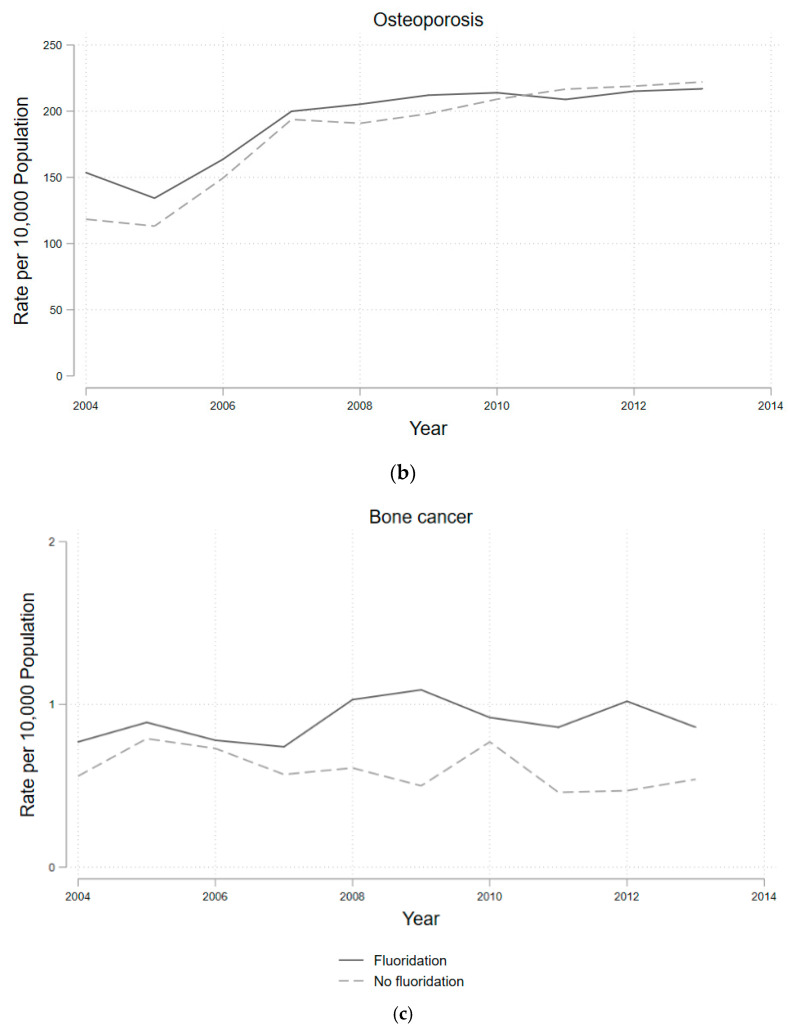
Age–sex adjusted standardized rates for three selected diseases: (**a**) hip fracture, (**b**) osteoporosis, (**c**) bone cancer from 2004–2013.

We plotted the age-standardized rates for the selected disease to observe its temporal trend over the period from 2004 to 2013 (Figure 2). For hip fracture and osteoporosis, the age-standardized rates tended to increase year to year, not only in fluoridated areas but also in non-fluoridated areas. We can clearly confirm that there was no increase particularly among fluoridated areas only. For bone cancer, it appeared to be slightly lower in non-fluoridated areas; however, this is due to the small number of patients.

A Bayesian spatio-temporal regression analysis was performed to calculate the posterior relative risk as shown in Table 2. The posterior relative risks of hip fracture, osteoporosis, and bone cancer were 0.95, 0.94, and 1.20, respectively. The fact that the relative risks of hip fracture and osteoporosis were less than 1.0 indicates that CWF did not increase the risk of adverse health effects. For bone cancer, the relative risk was slightly higher than that of the other two diseases because the number of patients was very small compared to other diseases. Comparing the relative risk value according to gender, for the fractures, males and females relative risk were 0.88 and 0.99, respectively. For osteoporosis, it was 0.86 and 0.95 for males and females, respectively. Both diseases (hip fracture and osteoporosis) were lower in males than in females. 

**Figure 3:**
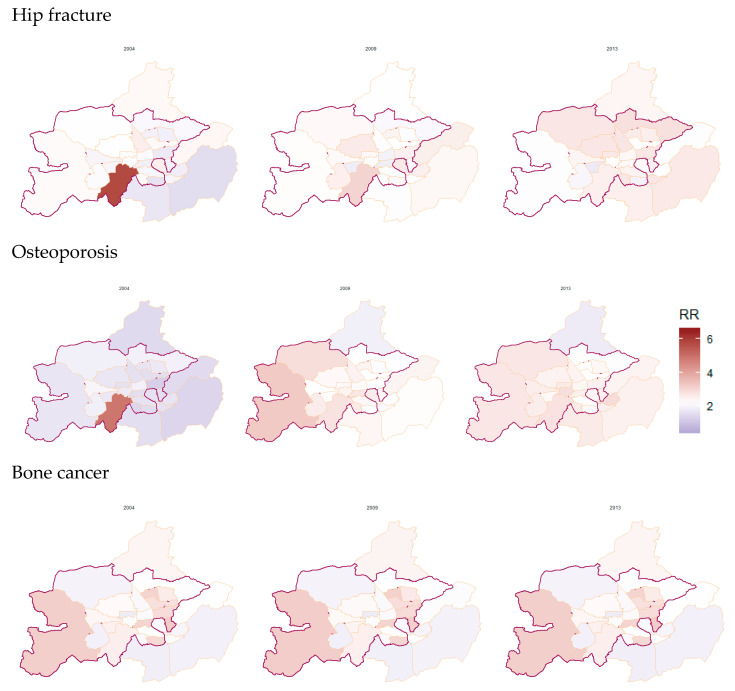
Spatial distribution of posterior relative risks by town and year in 2004, 2009, and 2013. Bold line: CWF was conducted, rest of regions: CWF never conducted.

Figure 3 provides information on the posterior distribution of relative risks for the selected diseases to show the spatial trend from 2004 to 2013 but we only presented three years (2004, 2009, and 2013) to see differences as time passed by. The area marked with a bold line is the region where CWF was implemented, and the rest of the area was classified as non-CWF area. Consistent with previous result, the spatial pattern of areas of the prevalence of selected diseases increased over time with a color change from purple to dark red. However, we observed that there was no significant difference between CWF and non-CWF areas in the three selected years (2004, 2009, and 2013) for hip fracture and osteoporosis. In the case of bone cancer, the CWF area has a higher relative risk than the non-CWF areas, but this is due to the low number of cases compared to the other two diseases.

In the present study, we showed the model performance to show the best-fitted model by comparing the DIC value between R-INLA method and CARBayesST in terms of sensitivity analysis. Each model was fitted to calculate DIC generated by an MCMC simulation, and in each case, 50,000 interactions were discarded as burn-in. By comparing DIC value, Model 4 had the lowest DIC value in hip fracture and osteoporosis, but it took a longer computational time. Consequently, Model 1 appeared to be the best choice overall in all three diseases since the DIC value was consistent with previous R-INLA results and it had reasonable computation time compared to other models (Supplementary Materials Table S1).

## 5. Discussion

In this study, we examined the association between drinking fluoridated tap water and selected bone diseases (i.e., hip fractures, osteoporosis, and bone cancer) using a natural experiment design in Cheongju, Korea. We compared the prevalence of selected diseases in residences from 2004 to 2013 in fluoridated and non-fluoridated areas in Cheongju with data collected from NHIS and MIDS data from Statistics Korea. We found that there was no clear evidence that exposure to fluoridated water increased health risks at the town level in Cheongju. These findings suggest that there is no clear evidence of adverse health effects occasioned by exposure to fluoridation. 

A large number of studies on the association between fluoride exposure and bone diseases have mainly focused on fractures. A meta-analysis of the results of these studies confirmed that chronic fluoride exposure (mainly from drinking water) does not significantly increase the risk of hip fractures [26]. In addition, a study conducted in the United States also concluded that long-term exposure to fluoridated drinking water does not increase the risk of fractures [27]. Furthermore, another study conducted in Ireland confirmed that there is no significant relationship between water fluoridation and bone health [28]. These findings corroborate the results of the present study, in which the posterior relative risk for hip fractures and osteoporosis was less than one. In addition, a 2018 England monitoring report concluded that there was no clear association between fluoridation and adverse health outcomes [29]. The role of CWF in preventing dental caries has been affirmed in previous studies that evaluated the systematic effect of CWF in Cheongju [13]. The present study only presents results on the association of CWF with hip fractures, osteoporosis, and bone cancer. The results showed that the relative risks for hip fractures and osteoporosis were significantly lower in men. Similarly, previous studies have shown that exposure to fluoride at a concentration of over 2 ppm lowers the rate of fractures [30]. Moreover, fluoride salt are used in male osteoporosis therapy to reduce the risk of fractures by increasing bone mass [31]. It is important to note that hormonal changes in men were not as significant as in women, and women have higher bone loss due to menopause, which is not equivalent to the loss of testosterone and reduction of estrogen levels with age in men [32]. This explains why the relative risk for hip fractures and osteoporosis was lower in men than in women. 

One of the key strengths of this study is that it was designed to be a natural experiment without the intervention of researchers, and it compared adverse health effects in the CWF and non-CWF regions. Internal comparisons within the same population group on a small area unit can provide clear evidence on the adverse health effects of CWF. Furthermore, the spatio-temporal analysis allowed us to observe the differences between CWF and non-CWF regions and the adverse health effects by visualizing the prevalence of the disease. 

Our study has a few limitations. First, it was difficult to determine how many residents were exposed to fluoridated water in CWF regions. Non-differential misclassification could occur because individual exposure statistics were unknown and, as a result, it might appear that there was no difference in prevalence between the two regions due to bias toward a null hypothesis. However, the rate of drinking tap water in both regions was almost the same (47.8% in CWF regions and 45.5% in non-CWF regions). This means that there were no major differences in the exposure to fluoride levels in either regions. Second, fluoride exposure should be long enough to facilitate an evaluation of the dose-response in human health in order to establish the link between fluoridation and a specific disease. However, in the present study, the percentage of people who lived in the investigated regions for more than 25 years was small (at 9.0% and 5.1%, respectively in Table 1).

Nevertheless, this did not affect the difference in the prevalence. Despite the difference in the proportion of people who have lived in both regions, it would have been difficult to observe the differences in the prevalence of bone diseases even though they truly have been exposed to fluoridation over a long period of time. Third, this study could not measure the individual level of fluoride exposure compared to the Brazilian study conducted using the same natural experimental design [33]. Given the types of water resources and drinking water, there was no difference in fluoride exposure between the two regions. Therefore, it might be challenging to ascertain whether the measurements were underestimated or overestimated. Fourth, since this study relied on bone disease claims data, and a single disease occurrence can result in limitations such as the use of multiple institutions or the accuracy of diagnosis names [19]. Furthermore, part of the definition of a hip fracture may also worsen specificity or have a greater effect on estimates of the sensitivity [34]. However, small areas in the region were analyzed using claims data to ensure that it did not affect the results.

Many studies have proved that CWF helps prevent dental caries and improve the general public’s dental health. Despite this, there is still opposition to fluoridation. This opposition has not persuaded many countries to stop implementing fluoridation projects because doing so would be a great loss for many nations and people, including social minorities, who rely on fluoride (because it is easily available and affordable) to maintain their oral health. In addition, opponents of fluoridation are vanguards of anti-scientific activities, such as anti-vaccination and anti-fluoridation, for similar reasons [10]. Therefore, if CWF is halted, some groups may increase pressure to stop vaccination programs, and other community-wide initiatives, leading to the failure of the public health system. 

Local governments are vulnerable to the voices of the opponents of CWF. Their opposition is witnessed all over the world, specifically in community water fluoridation projects. In Korea, the CWF is completely ceased, a crisis to public health. To re-establish the public health program, the Ministry of Health and Welfare needs to address the public’s concerns by publishing research articles in professional journals to inform to public. The participation of experts from each local government is also urgently needed as well. Finally, although the effectiveness and benefits of public health prevention programs are evident, the local government is forced to suspend specific policies and institutional supplementation that are absolutely necessary due to opposition from some groups. 

## 6. Conclusions

In conclusion, using a spatio-temporal Poisson regression analysis, we conclude that the implementation of the water fluoridation program in CWF areas did not increase the risk of adverse health effects, typically bone diseases (hip fracture, osteoporosis, and bone cancer) compared to areas where it was never implemented. The results of this study provide evidence of benefits and no harmful effects on humans. Finally, well-design epidemiological studies of adverse health effects and large case-control studies that are linked to the spatio-temporal methods are recommended. 

## Figures and Tables

**Table 1:** Distribution of general characteristics in community water fluoridation (CWF) and non-CWF regions from 2004 to 2013.

Variables	CWF		Non-CWF	
	No. of Residents	%	No. of Residents	%
Total	4,406,021		2,270,959	
Gender				
Male	2,200,104	49.9	1,126,495	49.6
Female	2,205,917	50.1	1,144,464	50.4
Age (years)				
<20	1,135,966	25.8	603,984	26.6
20–39	1,473,753	33.4	650,749	28.7
40–59	1,292,255	29.3	813,074	35.8
60–79	445,321	10.1	177,593	7.8
≥80	58,726	1.33	25,559	1.13
Population density (people per km^2^)	5.1		3.4	
Number of towns (N)	21		9	
Education level *				
Middle school or lower	1689	36.8	2541	34.9
High school	1433	31.2	2140	29.4
College or higher	1469	32.0	2607	35.8
Period of residence *				
>1 year	324	23.5	691	34.1
1–5 years	493	35.8	1002	49.4
5–10 years	397	28.9	462	22.8
10–25 years	362	26.3	461	22.7
≥25 years	124	9.01	104	5.13
Source of water *				
Community water system	4363	98.8	2681	98.6
Village water (temporal)	6	0.14	0	0.00
None	49	1.11	39	1.43
Types of drinking water				
Drinking tap water	812	47.8	1237	45.5
Purified tap water	524	30.9	860	31.6
Bottled water	238	14.0	427	15.7
Others	124,691	7.30	196	7.21

* indicate that the data obtained from MIDS of Statistics Korea, 2010 Korean Census.

**Table 2:** Posterior distribution of relative risks with 95% credible intervals.

	Total	Male	Female
	RR * (95% CrI **)		
Hip Fracture	0.95 (0.87–1.05)	0.88 (0.75–1.01)	0.99 (0.89–1.09)
Osteoporosis	0.94 (0.87–1.02)	0.86 (0.76–0.97)	0.95 (0.87–1.03)
Bone Cancer	1.20 (0.89–1.61)	1.26 (0.84–1.88)	1.03 (0.87–1.22)

* RR: Relative risk; ** 95% CrI: 95% Credible Interval.

## Supplementary Materials

The following are available online at https://www.mdpi.com/1660-4601/17/24/9170/s1, Table S1: The result of comparing models used by CARBayesST, Table S2: R-INLA coding, Table S3: R-CARBayesST coding.
