# Supplementary material for: The Association between Community Water Fluoridation and Bone Diseases: A Natural Experiment in Cheongju, Korea
Source: Int J Environ Res Public Health. 2020 Dec 9;17(24):9170. doi: 10.3390/ijerph17249170 (PMC7764285; doi:10.3390/ijerph17249170)
Supplement: Supplementary file 1 [file ijerph-17-09170-s001.pdf]

Table S1. The result of comparing models used by CARBayesST.

	Model	Hip fracture			Osteoporosis			Bone cancer		
		Time (s)	DIC	p_D *	Time (s)	DIC	p_D *	Time (s)	DIC	p_D *
**CARBayesST**	Model 1: CARanova	142.9	2118.5	171.8	147.1	3821.7	353.7	162.3	1029.3	29.4
	Model 2: CARlinear	132.2	2123.8	52.7	127.9	6163.7	65.3	141.1	1026.1	24.4
	Model 3: CARar	98.5	2102.4	151.6	102.8	3272.0	323.6	100.6	1011.2	42.3
	Model 4: CARadaptive	545.3	2007.7	74.2	478.9	2001.8	72.3	480.8	2004.9	72.0
	Model 5: CARlocalised	389.9	2029.2	109.6	374.3	2029.3	108.8	388.2	2027.5	109.3
R-INLA	Type 1 interaction	3.23	2100.1	159.7	2.76	3212.3	304.8	2.24	1021.7	21.9

We used the package called CARBayesST in R which is the first proprietary software package for spatio-temporal unit modeling with CAR prior (Lee et al., 2018). With the package, we based on Markov chain Monte Carlo (MCMC) to compare several models which applied the spatial temporal interaction and following models are selected; CARlinear CARanova, CARsepsatial, CARar, CARadaptive and CARlocalised.

Table S2. R-INLA coding.

```
> fom1_hfx = hfx_case ~ f(region, model='bym', graph=cheongju) +
                        f(time, model="rw2") + f(time1, model="iid") +
                        f(region.time, model="iid") +
                        flo
> result1_hfx= inla(fom1_hfx, family="poisson", data=dat, E=dat$hfx_exp,
                        control.compute=list(dic=TRUE, cpo=TRUE,
                        graph=TRUE, mlik=TRUE) )

> fom2_hfx = hfx_case ~ f(region, model='bym', graph=cheongju) +
                        f(time, model="rw2") + f(time1, model="iid") +
                        f(region.int, model="iid",group=time.int,
                        control.group=list(model="rw2")) +
                        flo

> fom3_hfx = hfx_case ~ f(region, model='bym', graph=cheongju) +
                        f(time, model="rw2") + f(time1, model="iid") +
                        f(time.int, model="iid", group=region.int,
                        control.group=list(model="besag", graph=cheongju)) +
                        flo

> fom4_hfx = hfx_case ~ f(region, model='bym', graph=cheongju) +
                        f(time, model="rw2") + f(time1, model="iid") +
                        f(region.int, model="besag", graph=cheongju,
                        group=time.int, control.group=list(model="rw2")) + flo
```

These R codes are for fitting each type of formula with hip fracture. We did not include other selected diseases (osteoporosis, bone cancer) because it also performed in the same way.

Table S3. R-CARBayesST coding.

```
> m1_hfx = CARBayesST::ST.CARanova(f_hfx, data=dat, W=W.mat, family="poisson",
           interaction=F, burnin=5000, n.sample=20000,thin=5)
> m2_hfx = CARBayesST::ST.CARlinear(f_hfx, data=dat, W=W.mat, family="poisson",
           burnin=5000, n.sample=20000, thin=5)
> m3_hfx = CARBayesST::ST.CARar(f_hfx, data=dat, W=W.mat, family="poisson",
           burnin=5000, n.sample=20000, thin=5)
> m4_hfx = CARBayesST::ST.CARadaptive(f_hfx, data=dat, W=W.mat, family="poisson",
           burnin=5000, n.sample=20000, thin=5)
> m5_hfx = CARBayesST::ST.CARlocalised(f_hfx, data=dat, W=W.mat, family="poisson",
           G=3,burnin=5000, n.sample=20000, thin=5)
```

These R codes are for fitting each type of formula with hip fracture. We did not include other selected diseases (osteoporosis, bone cancer) because it also performed in the same way.
